# Effect of complexing agents on the electrochemical performance of LiFePO_4_/C prepared by sol-gel method

**DOI:** 10.1186/1556-276X-7-40

**Published:** 2012-01-05

**Authors:** Rong Yang, Erwei Kang, Bailing Jiang, Jou-Hyeon Ahn

**Affiliations:** 1 Department of Chemical and Biological Engineering and Research Institute for Green Energy Convergence Technology, Gyeongsang National University, 900 Gajwa-dong, Jinju, 600-701, Korea; 2 Department of Applied Chemistry, Xi'an University of Technology, Xi'an, 710048, Shaanxi, People's Republic of China

**Keywords:** lithium-ion batteries, cathode material, lithium iron phosphate, sol-gel method, complexing agent

## Abstract

LiFePO_4_/C is synthesized via sol-gel method using Fe^3+ ^as iron sources and different complexing agents, followed by sintering at high temperature for crystallization. The amount of carbon in these composites is less than 6.8 wt.%, and the X-ray diffraction experiment confirms that all samples are pure single phase indexed with the orthorhombic Pnma space group. The particle size of the LiFePO_4_/C synthesized by acetic acid as a complexing agent is very fine with a size of 200 nm. The electrochemical performance of this material, including reversible capacity, cycle number, and charge-discharge characteristics, is better than those of LiFePO_4_/C synthesized by other complexing agents. The cell of this sample can deliver a discharge capacity of 161.1 mAh g^-1 ^at the first cycle. After 30 cycles, the capacity decreases to 157.5 mAh g^-1^, and the capacity fading rate is 2.2%. The mechanism is studied to explain the effect of a complexing agent on the synthesis of LiFePO_4_/C by sol-gel method. The results show that the complexing agent with a low stability constant may be proper for the synthetic process of LiFePO_4_/C via sol-gel method.

## Introduction

LiCoO_2_, LiNiO_2_, and LiMn_2_O_4 _have been widely used as cathode materials in lithium-ion batteries currently. More recently, LiFePO_4 _has become one of the most promising candidates due to its relative lack of toxicity, inexpensiveness, and its ability to provide a long cycle life and high rate at high temperatures [1,2]. LiFePO_4 _has a high lithium intercalation voltage of 3.4 V vs. lithium and a high theoretical capacity of 170 mAh g^-1^. However, it is difficult to make practical use of the full theoretical capacity at a high rate because the separation of the chain of FeO_6 _edge-shared octahedral contributes an extremely low electronic conductivity [3]. There are two drawbacks in using LiFePO_4 _as a commercial cathode material: one problem is its poor electronic conductivity, and the other one is the difficulty of synthesizing LiFePO_4 _because of iron oxidation state.

It is an effective method to improve the electrochemical property of LiFePO_4 _by supervalent ion doping [4,5], carbon coating [6,7], and synthesizing particles with well-defined morphology [8]. Olivine-type LiFePO_4 _can be synthesized by a solid-state reaction, sol-gel method, mechanical activation, or microwave heating, etc. The solid-state reaction has been widely adopted for synthesis of pure crystalline olivine-phase LiFePO_4_, resulting in the formation of large particles. Because the reaction occurred on a molecular level, sol-gel method is one of the effective means to prepare ultrafine particles [9-11]. Until now, most researchers have placed much more emphasis on the processing of the sol-gel method and its effect on the structures and electrochemical performances of cathode materials, but the mechanism is hardly studied to explain the effect of complexing agents in the process of preparation.

In this study, LiFePO_4_/C composites were prepared with different complexing agents by sol-gel method, and the analysis of the experimental data based on the ligand field theory was carried out. Tap density and charge-discharge experiments to evaluate the electrochemical performance of LiFePO_4_/C as a cathode material were also investigated.

## Experiment

Various LiFePO_4_/C cathode materials were synthesized by sol-gel method with acetic acid, ethanediol, oxalic acid, and ethylenediamine as complexing agents, and they were named as SGAc, SGEg, SGOc, and SGEn, respectively. The ingredient chemicals LiNO_3 _(A.R, Shanghai Chemical Reagents Co. Ltd., China), iron (III) nitrate Fe(NO_3_)_3_·9H_2_O (A.R, Shanghai Chemical Reagents Co. Ltd., China), and NH_4_H_2_PO_4 _(A.R, Shanghai Chemical Reagents Co. Ltd., China) were dissolved stoichiometrically in the saturated solution of complexing agent in water, and then, ascorbic acid was added to form a homogeneous solution after continuous stirring for 1 h at 55°C. The reaction mixture was adjusted to a pH of 6.5 with ammonia water and stirred at 75°C, and then, the solution was evaporated to dryness. The residue was formed into gel, which was dried in a vacuum drying oven at 80°C for 12 h. The mixture was pelletized and heated in a nitrogen atmosphere at 600°C for 4 h, and then cooled slowly to room temperature.

LiFePO_4_/C was characterized by scanning electron microscopy [SEM] and X-ray diffraction [XRD]. The SEM observation was performed on a JSM-5610 scanning electron microscope (JEOL Ltd., Akishima, Tokyo, Japan). X-ray diffraction was carried out by a Rigaku-D/MAX-2400 diffractometer (Rigaku Corporation, Tokyo, Japan) with CuKα radiation (*λ *= 0.15406 nm).

The testing electrodes were made by dispersing 80 wt.% active material, 15 wt.% acetylene black, and 5 wt.% poly(vinylidene fluoride) binder in a dimethyl phthalate solvent to form a slurry. The slurry was coated onto an Al foil and dried in a vacuum oven at 80°C. The separator was a Celgard 2400 microporous membrane (Celgard, LLC, Charlotte, NC, USA). 1 M LiPF_6 _solution in ethylene carbonate-dimethyl carbonate mixture (1:1 in volume) was used as an electrolyte, and lithium foil was used as an anode. The prototype cell was assembled in a dry glove box under argon. Cyclic voltammetry [CV] was performed at a scanning rate of 0.05 mV·s^-1 ^from 2.0 V to 4.5 V. The charge-discharge cycle performance was examined by an Arbin instrument BT-2000 (Arbin Instruments, College Station, TX, USA) between 2.5 and 4.3 V vs. Li^+^/Li at room temperature.

## Results and discussion

**Figure 1:**
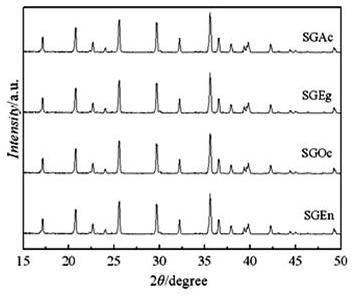
**XRD patterns of LiFePO_4_/C synthesized by sol-gel method with different complexing agents**.

XRD patterns of the samples prepared with different complexing agents were shown in Figure 1. All the samples were pure single phase indexed with the orthorhombic Pnma space group. No evidence of diffraction peaks for crystalline carbon (graphite) appeared in the diffraction pattern, which indicated that the carbon generated from the complexing agent was amorphous and that its presence did not influence the structure of LiFePO_4_/C. The carbon content of the samples determined by element analysis was 2.3, 4.8, 3.2, and 5.1 wt.% for SGAc, SGEg, SGOc, and SGEn, respectively.

**Figure 2:**
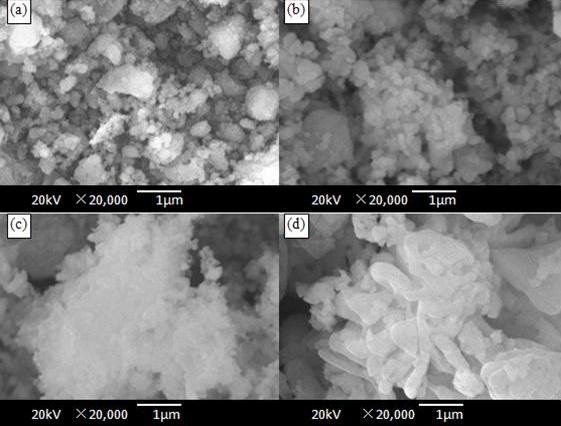
**SEM images of LiFePO_4_/C samples**. (**a**) SGAc, (**b**) SGOc, (**c**) SGEg, and (**d**) SGEn.

The morphology for LiFePO_4_/C powders was observed on SEM, as shown in Figure 2. The SCAc consisted of non-uniform fine particles with a size range of 100 nm to 1 μm, as shown in Figure 2a. We found that big particles were secondary particles composed of small size particles (around 100 nm). The unsolid aggregation state had no effect on the permeation of electrolyte between particles, and it benefited to the improvement of tap density. The particle size of SCOc was about 100 to 200 nm, as shown in Figure 2b. The small particle size of LiFePO_4_/C would be beneficial in reducing the diffusion length of the lithium ion inside, resulting in fast reaction and diffusion kinetics, and also benefited to the coating technique of cathode in the industrial production. There was a serious agglomeration in SCEg, and the particle of SCEn was layered as thin film structures and was larger than others.

**Figure 3:**
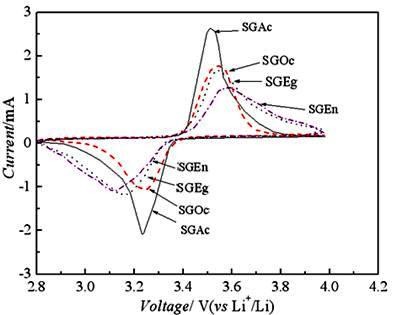
**Cyclic voltammetry curves of LiFePO_4_/C**.

Cyclic voltammetry measurement was performed on the LiFePO_4 _electrode at a scanning rate of 0.05 mVs^-1^. Figure 3 showed the cyclic voltammogram of LiFePO_4_/C with different complexing agents. The anodic and cathodic peaks of SGAc appearing in the CV curves were sharp and were of better symmetry. The difference between the anodic and cathodic peaks was smaller compared to others. It was shown that the sample had good electrode reaction kinetics in the cell.

**Figure 4:**
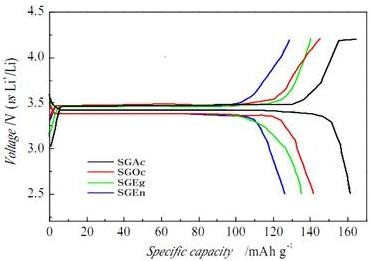
**Initial charge-discharge profiles of LiFePO_4_/C**.

Initial charge-discharge profiles of LiFePO_4_/C synthesized by sol-gel method with different complexing agents were shown in Figure 4. All samples demonstrated a charging and discharging plateau. The potential for charging is approximately 3.49 V, and the discharging potential is approximately 3.37 V, which is entirely consistent with the result from the CV test. The small voltage difference between the charge-discharge plateaus represents their good kinetics. The initial discharge capacity of SGAc showed 161.1 mAh g^-1 ^and was higher than that of other samples.

**Figure 5:**
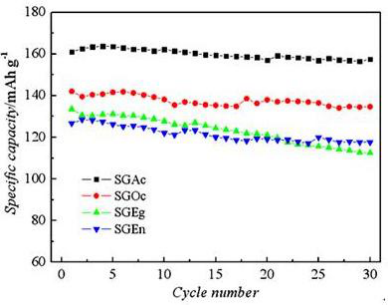
**Cycle life of LiFePO_4_/C**.

The cycling performance of these samples was shown in Figure 5. The cell of sample SGAc can deliver a discharge capacity of 161.1 mAh g^-1 ^at the first cycle. After 30 cycles, the capacity decreased to 157.5 mAh g^-1^, and the capacity fading rate was 2.2%.

From the above analysis, we can conclude that the electrochemical performance of LiFePO_4_/C prepared by sol-gel method was affected by the complexing agents. Therefore, we also explored the possible effect mechanism of a complexing agent on the synthesis of LiFePO_4_/C by sol-gel method.

In the process of the sol-gel method, gel was formed by a reaction between the Fe^2+ ^and complexing agent. There were three states which were sol, gel, and sintering products in the whole process. Because the electrochemical properties of LiFePO_4_/C depended on the particle size and its homogeneity, it was important that the gel was formed. The difference among gels was formed by using different complexing agents, so the effect mechanism of complexing agents on the synthesis of LiFePO_4_/C would be explained by coordinate chemistry theory.

The stability of complex compounds formed by different complexing agents and metal ions was varied. Table 1 listed the cumulative stability constant of Fe^2+ ^with various complexing agents and the discharge specific capacity, tap density, and carbon content of LiFePO_4_/C synthesized by sol-gel method.

**Table 1:** Effects of complexing agents on Fe^2+ ^and the characteristics of LiFePO_4_/C synthesized by sol-gel method

Complexing agent	Stability constant (log*β*)	Discharge specific capacity (mAh g^-1^)	Tap density (g cm^-3^)	Carbon content (wt.%)
Acetic acid	8.3	161.08	1.37	2.3
Ethanediol	9.67	133.53	1.16	4.8
Oxalic acid	5.22	143.04	1.23	3.2
Ethylenediamine	9.70	126.73	1.12	5.1

Stability constant of Fe^2+ ^with various complexing agents and the discharge specific capacity, tap density, and carbon content of LiFePO_4_/C synthesized by sol-gel method.

According to Table 1, we can see that the discharge specific capacity of the material that was formed by the acetic acid monodentate ligand was 161.1 mAh g^-1^, which was the highest. The discharge capacity of the material that was formed by different complexing agents had a reverse relationship between the stability constant of Fe^2+ ^and various complexing agents. That is, the higher the stability constant, the lower the discharge capacity. Based on coordinate chemistry theory, complexes with a larger stability constant are more stable, and those complexes are difficult to decompose when sintering. The final product preserved the multi-ring interleaving structure, to a great extent, that lead to the poor crystallinity and low discharge capacity of the product. However, complexes with a low stability constant were unstable, and those complexes were easy to decompose when sintering at the same temperature. The average particle size of the final product was small, which was beneficial in reducing the diffusion length of the lithium ion inside, resulting in fast reaction and diffusion kinetics, which can increase the electrochemical properties of material.

However, we also found that there was one exception against the above principle. The discharge capacity of the material formed by the acetic acid monodentate ligand is 161.1 mAh g^-1 ^which was the highest, while the stability constant, log*β*_*3*_, of Fe^2+ ^with acetic acid (8.3) was larger than that of oxalic acid (5.22). By further analysis, we found that ethanediol, oxalic acid, and ethylenediamine were bidentate ligands. By coordinating with bidentate ligands, Fe^2+ ^was in an octahedral field. For the energy grade split of *d *orbits, the *d *electron configuration of the high-spin Fe^2+ ^in an octahedral field was *t*_2g_^4^*e*_g_^2^, and the pairing electron number was 1.So, its crystal field stabilization energy [CFSE] was

$$\text{CFST}\left(\text{o}\right)=n_{1}E\left(t_{2g}\right)+n_{2}E\left(e_{g}\right)=n_{1}\left(-4D_{q}\right)+n_{2}\left(6D_{q}\right)=4\left(-4D_{q}\right)+2\left(6D_{q}\right)=-4D_{q}$$

where *n*_1 _was electron number in *t*_2g _orbit, *n*_2 _was electron number in *e*_g _orbit.

Acetic acid was a monodentate ligand; Fe^2+ ^coordinated with three acetic acid molecules and one water molecule at the same time; so, Fe^2+ ^was in a tetrahedral field composed of three oxygen atoms provided by acetic acid and one oxygen atom provided by water. The *d *electron configuration of the high-spin Fe^2+ ^in a tetrahedral field was *e*_g_^3^*t*_2g_^3^, whose pairing electron number was one.

So, its CFSE was

$$\text{CFST}\left(\text{t}\right)=n_{1}E\left(t_{2g}\right)+n_{2}E\left(e_{g}\right)=3\left(1.78D_{q}\right)+3\left(-2.67D_{q}\right)=-2.67D_{q}$$

The CFSE that high-spin Fe^2+ ^gained from the tetrahedral field was lower than that from the octahedral field. It is said that the stability Fe^2+ ^in the tetrahedral field was poorer than that in the octahedral field, so the discharge capacity of the material formed with acetic acid was higher than that formed with oxalic acid.

In the process of complex decomposition, the carbon would partially produce residues in the product. The carbon content of LiFePO_4_/C had a direct ratio to the stability constant of Fe^2+ ^with various complexing agents. The trend was consistent with the rule that the lower the stability constant was, the easier the product decomposed.

In short, complexing agents with a small stability constant would be chosen to format the precursor of LiFePO_4_/C, which would decompose easily due to poor stability when heating. Such method would beneficial in improving the electrochemical properties of the material.

## Conclusions

The LiFePO_4_/C was synthesized via sol-gel method by using Fe^3+ ^as iron sources, ascorbic acid as reducing reagent, and acetic acid, ethanediol, oxalic acid, and ethylenediamine as complexing agents. The LiFePO_4_/C synthesized with acetic acid as complexing agent has better electrochemical performance, having a discharge capacity of 161.1 mAh g^-1 ^at the first cycle, and the capacity decreases to 157.5 mAh g^-1 ^after 30 cycles. The capacity fading rate is 2.2%. According to the crystal field theory, a complexing agent with a small stability constant would be decomposed easily due to poor stability when heating. However, we found that the discharge capacity of LiFePO_4_/C synthesized with acetic acid as complexing agent was not consistent with the principle. The problem was solved by computing the CFSE of Fe^2+ ^in a crystal field.

## Competing interests

The authors declare that they have no competing interests.

## Authors' contributions

RY carried out the experimental works on SEM, XRD, cycle testing, etc. EK participated in the cycle testing. BL participated in the design of the study. JHA participated in drafting the manuscript. All authors read and approved the final manuscript.
